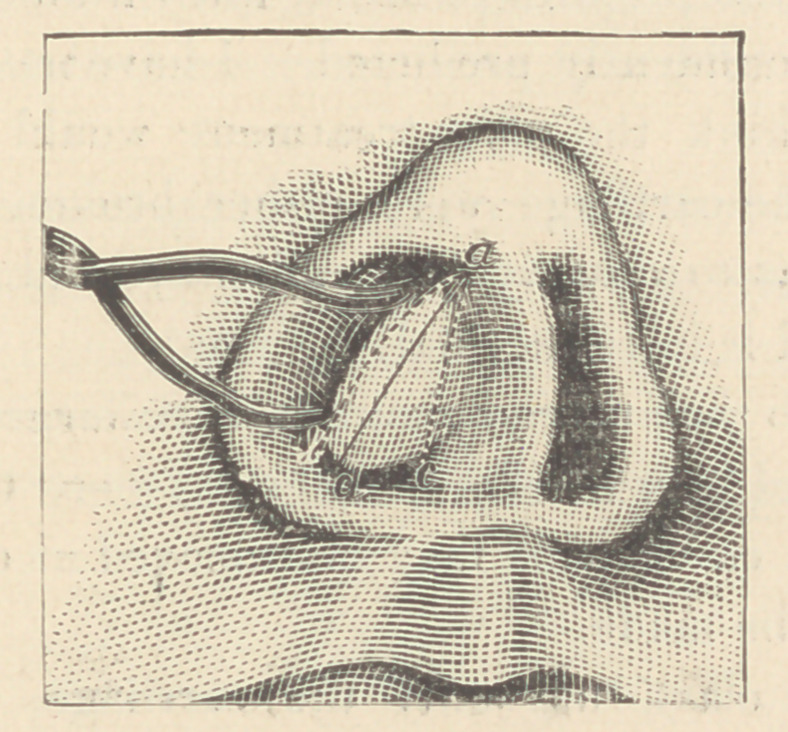# Deflection of the Septum Narium*Read before the American Laryngological Association, session 1882. Reprinted from the Archives of Laryngology, Vol. III, No. 4, Oct. 1882.

**Published:** 1882-12

**Authors:** E. Fletcher Ingals

**Affiliations:** Professor of Diseases of the Throat and Chest, Woman’s Medical College; Lecturer on Diseases of the Chest and Laryngology, Rush Medical College, Chicago


### Selections.

---

DEFLECTION OF THE SEPTUM NARIUM.* By E. FLETCHER INGALS, M.D., Professor of Diseases of the Throat and Chest, Woman's Medical College; Lecturer on Diseases of the Chest and Laryngology, Rush Medical College, Chicago.

Deflection of the nasal septum consists of a bending to one side either of the cartilaginous or bony septum, or of both. It is characterized by more or less obstruction of the naris and deformity of the nose. A slight degree of deflection is very common; indeed, it is so often found that it is not considered a pathological condition; but a degree of distortion sufficient to materially interfere with respiration, and to cause more or less deformity of the nose, is frequently met with. It is to these latter cases that I wish to direct attention.

For the sake of convenience in description, I will divide the cases into four classes: 1. Those in which there is slight bending of the whole septum. 2. Those in which there is bending of the septum, and more or less depression of the nose, due to injury. 3. Those in which there is local flexion, near the nostril, of the cartilage only. 4. Those in which a ridge of considerable size runs upward and backward, from near the nostril, along the line of articulation of the vomer with the cartilage, which ridge is formed mostly of bent cartilage, but partly of the bent vomer.

Judging from my own observation, the affection usually occurs in early life, either before or about the age of puberty.

---

* Read before the American Laryngological Association, session 1882.

Reprinted from the Archives of Laryngology, Vol. III, No. 4, Oct. 1882.

This deflection may be of traumatic origin, but usually it appears to be spontaneous. Some patients attribute it to an accident; such, for example, as being thrown from a horse, or falling upon the ice; but in the majority of cases no cause is assigned, or the one given does not satisfactorily account for the condition. In searching for the cause of this affection, we usually find that the patient has been troubled with catarrhal symptoms for some time before obstructed respiration has been noticed, and this is the only symptom which is found with any degree of uniformity in the early history of these cases. Again, in many persons who are affected with chronic coryza, we find considerable distortion of the septum, particularly of its cartilaginous portion. Both of these facts point to inflammation of the nasal mucous membrane as one of the principal factors in the production of the deformity.

I have had no opportunity to observe post-mortem appearances, but from a study of clinical cases, and from the examination of numerous skulls, I am convinced that the affection of the septum usually commences in the cartilaginous portion, and that the flexion of the vomer, which often exists, is of mechanical origin, due to firm articulation of this bone with the cartilage.

We must bear in mind that we have a vertical septum, resting upon an unyielding base, and held firmly down by the nasal bones and soft tissues of the nose, therefore no addition can be made to its edges without causing flexion. In cases of spontaneous origin, the principal changes appear to have been in the edges of the cartilage, increased growth from which has caused it to bend upon itself; but in some cases there is also considerable thickening of the septum.

The process, I believe, generally depends upon congestion of the overlying mucous membrane, but its exact nature has not been determined. It is probably a simple hyperplasia of the cartilaginous cells, due to hypernutrition; but it may possibly be of the nature of rachitis, the pathology of which is obscure.

Sir W. Jenner* says the alterations in the bones in rachitis consist in an increased preparation for ossification, but an incomplete performance of the process.

* "Green's Pathology and Morbid Anatomy," 3d Am. ed., p. 222.

P. Henry Green * states that the zone of cartilaginous tissue which in health is being transformed into bone is very thin, but in rachitis it is greatly increased, particularly at the ends of the bones, but also beneath the periosteum.

It would seem, therefore, that at least one step in the development of rachitis consists in an abnormal growth of cartilage, and it is possible that the same conditions operate to enlarge the cartilaginous septum that causes the increase in rachitis. However, if we accept the theory that rachitis is due to checking of nutrition, it would appear inappropriate to class deflections of the septum as one of the manifestations of this disease; for in patients in whom deflection is observed, there is seldom any evidence of imperfect nutrition, and thus far I have not seen one presenting other signs of rachitis.

Patients with deflection of the septum generally apply for relief, because of obstructed respiration or deformity of the nose. But we usually find, also, that there is excessive secretion from the nasal mucous membrane, most of which passes behind the velum into the throat. We also observe that the voice has something of a nasal twang, but the progress of the flexion has usually been so gradual that the patient himself does not appreciate the change.

Wnen distortion of the septum is extreme, the point of the nose is often pushed to the opposite side, so as to cause considerable deformity, especially in ladies. I have found this deformity so often, that I am led to think that in a majority of cases, a crooked nose is the direct result of enlargement and deflection of the septum.

Upon inspecting the naris on one side, we find a free space, with more or less concavity of the septum, along which a narrow furrow will often be seen, corresponding to the line of greatest flexure. In the opposite naris we find convexity of the septum, varying in extent and position in different patients, but always greater than would at first be expected from examination of the larger naris.

Contrary to the experience of others, I have observe this convexity most frequently on the right side.

* Ibid.

In the first class of cases, there is a uniform concavity of one side, with a corresponding convexity of the other.

In the second class, the deformity depends upon the severity of the injury, and may consist of simple depression and bending of the septum, or of this conjoined with fracture of the nasal bones.

In the third class, there may be only a slight flexion of the cartilage, near the nostril, both the anterior and posterior extremities of which may be easily seen; but in many instances the bent septum passes obliquely downward, from its natural position above, to the ala, and then bends sharply upon itself, so as to lie almost horizontally across the nostril. In this form of distortion, the nostril is often found to be completely closed, and the point of the nose is crowded considerably to the opposite side. In such cases, thorough inspection may be impossible, but by drawing the ala well outward, we may usually obtain a view of the deeper parts of the naris.

In the fourth class, the deformity usually commences at the middle or upper third of the septum, and passes from that point outward and downward, nearly to the floor of the naris. It then bends sharply upon itself, forming a longitudinal ridge, which stands out from the normal plane from three to eight millimeters. The ridge thus formed generally passes obliquely upward and backward, a distance of two or three centimeters, in a line corresponding to the articulation of the vomer with the cartilage and nasal plate of the ethmoid.

The most prominent part of the flexure is usually found about two millimeters below the normal position of the junction of the nasal cartilage with the vomer. In these cases, though upon first inspection the inferior plane of the bent septum appears to rest upon the floor of the nasal cavity, it will generally be found that a space remains beneath it sufficient to allow the passage of a probe two or three millimeters in diameter.

There can be no difficulty in making a diagnosis if both nostrils are inspected, but it is sometimes impossible to determine the extent of the inflexion, in the deeper parts of the naris, until the cartilage in front has been removed.

In cases of spontaneous origin, the process of flexion continues for a limited, though uncertain time, but probably, in most cases, for at least two years. It finally comes to a standstill, and there seems no tendency to recurrence of the active increase in the size of the cartilage, neither is there likely to be any atrophy of it; but the deformity, unless relieved by an operation, will continue through life.

In traumatic cases, considerable flexion results immediately from the injury, but, judging from the extent of distortion in cases of this variety which have fallen under my observation, the injury is usually followed by considerable hypertrophy, and subsequent bending of the cartilage.

Several operations have been recommended for the relief of this condition. The most important are, the one recommended by S. D. Gross, which I believe is the same as that proposed by Chassaignac, which consists in paring off a portion of the bent septum; the operation proposed by Wm. Adams, for forcible replacement of the bent and depressed septum; and Goodwillie's operation for perforating the septum.

Several modifications have been made in each of these operations, which have better adapted them to special cases.

In the first class of cases, often no operation is necessary, though perforation by Steel's instrument, or Adam's operation, would probably correct the deformity, yet, even in these, I should prefer the removal of a slender triangular piece from the lower margin of the septum.

In the second class of cases, Adam's operation,* or the operation suggested by Dr. A. J. Steel, of St. Louis, and subsequently practiced by Dr. Glasgow,† seems most likely to be followed by favorable results, especially if there is no enlargement of the cartilage, in which case, if replaced, it is of exactly the proper dimensions to restore the nose to its former shape.

In the third and fourth classes, where there is an increased growth of cartilage, no operation is likely to be permanently successful which does not include the removal of the redundant tissue.

---

* *British Medical Journal*, Oct. 2, 1875.

† *St. Louis Courier of Medicine*, 1879; also, Transactions of Am. Laryngological Association, 1881, p. 117.

Simple perforation of the septum is not sufficient in these cases, for, although it will doubtless partially relieve the difficulty in respiration, by allowing air to pass from the obstructed nostril through the enlarged naris, it will not correct the deformity of the nose. It will not usually cure the obstruction in the back part of the occluded naris, and therefore it cannot cure the catarrhal symptoms; besides, as suggested at the last meeting by Dr. Glasgow, there is a tendency to scabbing of the edges of the perforation, and deformity of the nose is liable to result from removal of large portions of the septum.

Paring off portions of the septum will answer the purpose in some cases, but it is only suitable for a small number. By this operation, a thin septum would be left of the original size, and of insufficient strength to support the nose. Such a septum would be peculiarly liable to further distortion; consequently, in many cases, the operation would not only fail to correct the twisting of the nose, but it would favor subsequent depression of the external parts. The operation suggested by Dr. Jarvis I have not tried, and as it is to be fully discussed in the next paper, I will not comment upon it.

In all cases where the cartilage is much enlarged, the operation which seems to me most suitable, and which I have on two occasions found very satisfactory, consists in separating the mucous membrane and removing the redundant tissue, after which the mucous membrane is stitched down, and the septum retained in proper position until firm union has taken place.

The operation will sometimes require considerable time, and therefore in most instances an anæsthetic will be necessary.

Ether or chloroform may be employed, but nitrous oxide is preferable for short operations, such, for example, as that to be recommended for cases of the fourth class.

Before commencing the operation, when possible, a small Eustachian catheter should be carried through the obstructed nostril to the pharynx, and through it a catgut or a well-waxed silk ligature should be passed, for tamponing the posterior naris. This ligature should be attached to a suitable tampon of cotton or sponge, and tied in such a manner that about two inches of the end of the ligature may hang down the pharynx when the tampon has been placed in position. This hanging end will greatly facilitate the removal of the tampon after the operation. It is usually best to introduce the tampon before the anæsthetic is administered.

When the bent septum lies nearly horizontally across the nostril, the mucous membrane over the septum should be incised from above downward and outward, near the center of the nostril, as indicated by the line *a d* in drawing, and then, with the handle of a scalpel, or with delicate curved spuds, the membrane should be separated from all that portion of the septum which is to be removed.

Estimating carefully the amount of tissue which it will be necessary to resect in order to secure symmetry of the nose and a straight septum, the cartilage should now be cut through from the upper to the lower outer angle of the obstructing portion *a b*, as indicated by the dotted lines in the drawing, and then along the inner border from above downward, *a c*. With a little care, this incision can be made without cutting through the mucous membrane in the opposite naris. The cartilage may now be seized with forceps by the angle (*a*) and drawn downward and cut off. If the flexion extends backward along the septum, a slender triangular piece must also be removed from its lower border by means of scissors, a knife, or a short cutting hook.

The incisions should be so planned that when the septum is pushed into its normal position, the cut edges of the cartilage will be in apposition. The mucous membrane is now drawn down and stitched, thus holding the cartilage in proper position; but it must also be supported by a plug, or by a pledget of cotton, until firm union has taken place. I have used cotton for this purpose, but the plugs recommended by Adams and Glasgow might possibly answer a better purpose. After a few days, a tubular instrument of similar form would probably be more comfortable.

The twisting of the end of the nose will be found to have been remedied by replacing the cartilage, and if proper care is exercised to keep the septum in position, until union is firm, a perfect result may be confidently predicted. I have not made the experiment, but I think the after-treatment would be more easily carried out if the cartilage were either broken or incised at its upper part, so as to destroy its resiliency, and thus require less pressure to hold it in its normal position.

In addition to the anterior obstruction, a large ridge sometimes runs upward and backward a distance of two or three centimeters, as in the fourth class; this may be removed at once, or a second operation may be made.

In the fourth class, not only the cartilage, but usually the upper border of the vomer also, is bent; were it not for this, the operation might be readily performed by separating the mucous membrane, and then paring off the cartilage with a slender probe-pointed knife. On account of the bony tissue which has to be cut through, a slender saw will be found useful in operating on these cases. I have used a slender metacarpal saw, but I am now having made a much smaller instrument, which may be worked either by hand or by a dental engine, and which will, I think, materially facilitate the operation, and possibly render an anæsthetic unnecessary in cases of this class.

In these cases, the mucous membrane should be divided perpendicularly at the anterior extremity of the ridge, so as to allow the introduction of a spud; also along the lower angle of the deflected cartilage, at a point which will insure enough tissue to cover the cut surface of the septum. The membrane having been separated from the septum, the saw is carried beneath the projecting portion, close to the ridge of the maxillary bone, and the cut is made directly upward until the bone and cartilage are divided. The piece being removed, the mucous membrane falls down upon the cut surface, and may be stitched at its anterior extremity to the membrane covering the crest of the maxillary bone.

It is not often necessary to bend the septum after this operation, but if the flexion at its upper part is very great, it is advisable to restore it to its normal position, as after the operation for cases of the third class.

P. S. Since the above was printed, I have operated on a case twenty years of age, in which the bending of the septum was so great as to obstruct both nostrils. The deformity was first noticed during infancy.

The patient made a good recovery, and the deformity of the nose was much benefited, though, on account of the bending of the nasal bones, it could not be entirely corrected without a fracture of the latter, which was not deemed best. The obstruction of the naris was perfectly relieved, and the patient's general health improved. E. F. I.

## Figures and Tables

**Figure f1:**